# N-chlorotaurine does not alter structural tendon properties: a comparative biomechanical study

**DOI:** 10.1007/s00402-025-05851-7

**Published:** 2025-04-05

**Authors:** Armin Runer, Friedemann Schneider, Karl Wawer, Kerstin Gruber, Rohit Arora, Markus Nagl, Werner Schmoelz

**Affiliations:** 1 Medical University of Innsbruck, Dept. of Orthopedics and Trauma Surgery, Innsbruck, Austria; 2 Technical University of Munich, Dept of Sports Orthopedics, Munich, Germany; 3 Medical University of Innsbruck, Institute of Hygiene and Medical Microbiology, Innsbruck, Austria

**Keywords:** N-chlorotaurine, Vancomycin, Septic arthritis, Anterior cruciate ligament reconstructions, ACLR

## Abstract

**Introduction:**

N-chlorotaurine (NCT) is a well-tolerated antiseptic with broad-spectrum microbicidal activity and could therefore be a promising alternative to vancomycin, the current standard of care for the prevention of postoperative septic arthritis (PSA) after anterior cruciate ligament reconstruction (ACLR).

**Materials and methods:**

The aim of this study was to evaluate whether soaking bovine extensor tendons in N-chlorotaurine (NCT), vancomycin, or 0.9% saline influences structural tendon properties. In this controlled biomechanical study, fifty bovine extensor tendons were randomized into groups and soaked for 10 min in distilled water solutions containing either 1% vancomycin, 1% NCT, 5% NCT, 5% NCT with 0.1% ammonium chloride, or 0.9% saline. Tendons were then mounted in cryo-clamps and subjected to uniaxial tensile testing until failure. Failure mode, ultimate load, ultimate elongation, and stiffness of the linear region from the load-elongation curve were extracted and compared for each graft.

**Results:**

No statistically significant differences were detected across all measured parameters (*p* > 0.05) and solutions. The mean ultimate load, ultimate elongation, stiffness and elastic modulus were not statistically significantly different between all five tested solutions.

**Conclusions:**

Both NCT and vancomycin even at high concentrations do not impair structural tendon properties compared to 0.9% saline. NCT appears to be safe for clinical use from a biomechanical perspective.

## Introduction

Postoperative septic arthritis (PSA) is a rare yet severe complication after anterior cruciate ligament reconstructions (ACLR) with a reported incidence between 0% and 2.1% [1]. Specific factors such as the type of graft used and patient demographics can influence this rate; for instance, the use of hamstring tendon autografts has been associated with a higher incidence of PSA. PSA significantly impacts patient outcomes and contributes to increased healthcare costs due to repeated surgical interventions, extended hospital stays, and the necessity for prolonged intravenous (i.v.) antibiotic therapy [1, 2]. In efforts to reduce the incidence of PSA, vancomycin—a potent glycopeptide antibiotic effective against gram-positive bacteria—has been widely adopted for prophylactic use [3]. ACL grafts are typically wrapped in vancomycin-saturated sterile gauze before implantation [3–6]. Consequently, PSA rates have markedly decreased to under 2% in recent years [3, 6–8]. Despite the promising clinical outcomes associated with vancomycin use, there remains a lack of comprehensive understanding regarding its toxicity profile, potential long term adverse effects, latent allergic reactions, and the risk of resistance development [9]. Additionally, conflicting data exists concerning the chondrotoxic effects of intra-articularly applied vancomycin [10–13]. In addition to chondrotoxicity, the question of the influence on the structural properties of ACL grafts remains. Current studies suggest that vancomycin does not detrimentally impact the biomechanical properties of tendons, such as maximum load to failure or stiffness [14]. While vancomycin has clearly demonstrated efficacy as a topical agent for infection control in ACLR, there is a notable scarcity of evidence exploring alternative antimicrobial options.

A promising alternative to vancomycin may be N-Chlorotaurine (NCT), a potent and well-tolerated antiseptic of endogenous origin. NCT, the N-chloro derivative of the amino acid taurine, is synthesized by activated human granulocytes and monocytes during the oxidative burst via the NADPH oxidase and myeloperoxidase pathways [15–17]. It is thought to be involved in the inactivation of invading pathogens and in the termination of inflammation due to its anti-inflammatory activity [15, 16]. NCT exhibits broad-spectrum antimicrobial activity, effective against bacteria, viruses, fungi, and protozoa, while remaining safe for use on sensitive tissues such as the eyes, skin, ulcers, and the ear [18, 19]. As an endogenous product, NCT breaks down into natural products– primarily taurine and chloride - and does not undergo systemic distribution [20]. Given these attributes, NCT has significant potential as an alternative to topically applied antibiotics in ACL surgery.

This study aimed to evaluate the impact of varying concentrations of NCT on the structural properties of bovine extensor tendons, comparing the results to those of vancomycin and 0.9% saline solution. It was hypothesized that there would be no difference in structural tendon properties following the application of different NCT concentrations when compared to vancomycin or saline solutions.

## Materials and methods

### Preparation of the tendons

Fifty fresh frozen bovine ankles were obtained from the local abattoir. All animals were raised for slaughter and had a mean age of 24 months. The extensor tendons were carefully dissected at its full length and cut to a uniform length of 13 cm. The cross-sectional area (CSA) was measured at the mid-point of each graft under a minimal load to remove slack from the specimen using a clinical sizing block (AR-1886, Arthrex Inc. Naples, Florida, USA). Subsequently, the weight of the tendon was measured on a scale (Kern KB 2000–2 N, weighing capacity 2000 g, accuracy 0.01 g). Each tendon was then wrapped in a moist gauze, sealed airtight and stored at -20 °C. Ten hours prior to testing, tendons were removed from the freezer and allowed to slowly thaw at room temperature. Each tendon was then randomly assigned to one of the five testing groups using block randomization. Prior to biomechanical testing, each tendon was pre-soaked for 10 min in a gauze containing the respective solution. All tests were conducted at room temperature, and the tendons were kept moist with saline solution throughout the testing process to prevent desiccation.

### Preparation of the reagents

NCT was obtained as a crystalline, white sodium salt and stored at -20 °C until use [18]. It was freshly prepared just before testing each graft. Three different NCT solutions were created using 50 ml of distilled water: 1% NCT (NCT1), 5% NCT (NCT5) and 5% NCT with additionally 0,1% ammoniochloride (NH_4_Cl) (NCT5AC). Adding NH_4_Cl has been shown to enhance NCT’s activity, potentially increasing its clinical relevance for specific indications [18, 19]. Additionally, a 1% vancomycin solution was prepared according to clinical standards by dissolving 500 mg vancomycin in 50 ml of distilled water. For the control group, a solution of 50 ml of 0.9% physiological saline was utilized.

### Testing procedure

**Fig. 1:**
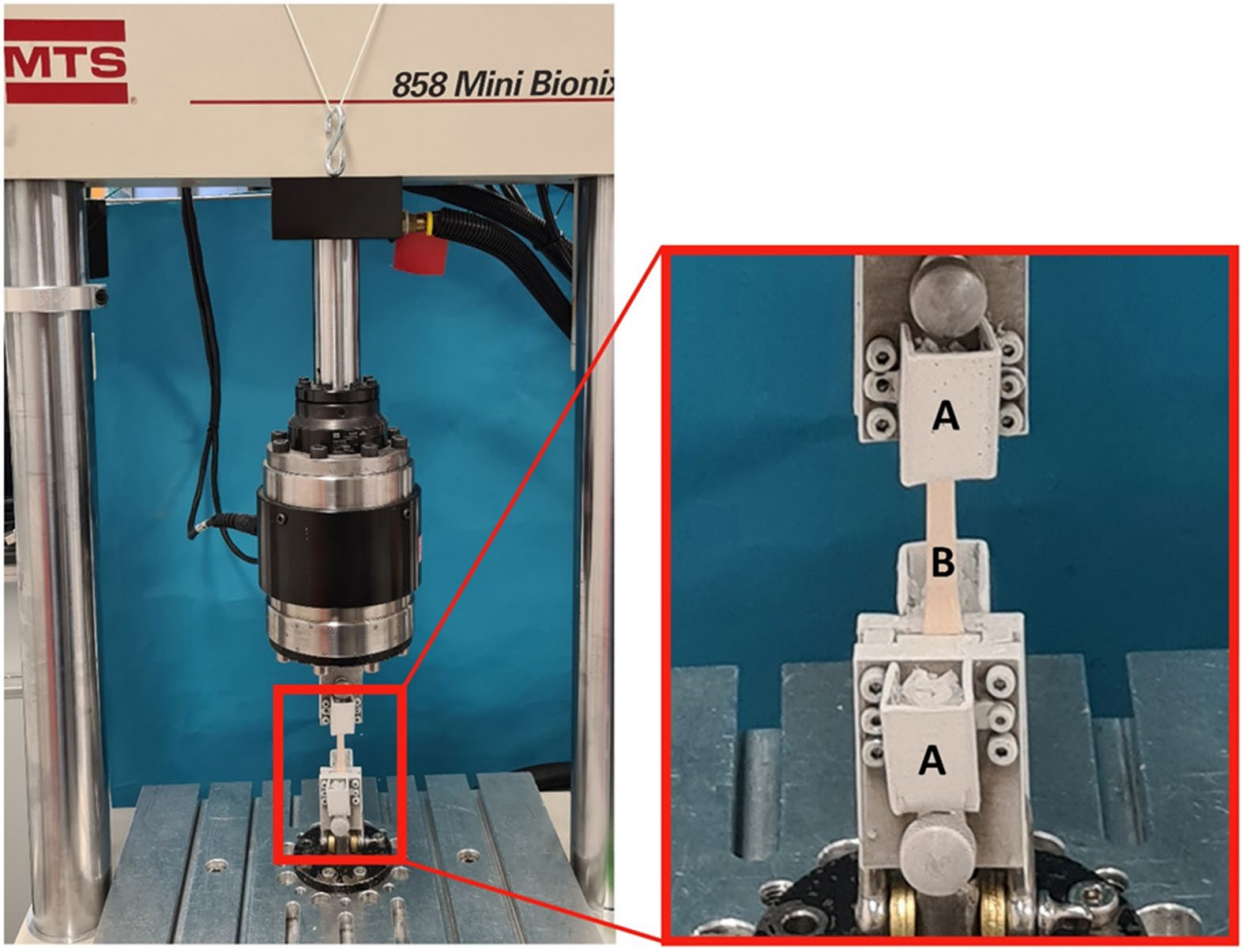
Material testing machine with setup for tensile testing and close up of cryo-clamp with dry-ice containers (**A**) and clamped tendon (**B**)

Tensile testing was performed using a uniaxial universal testing machine (MTS 858 Mini Bionix II, Minneapolis, USA, Fig. 1) under standardized laboratory conditions. Tendon fixation was achieved using a cryo-clamp equipped with dry-ice containers. Four centimeters of each tendon end were rigidly secured in the clamps, leaving a 5 cm segment of tendon exposed between the clamps. The dry-ice containers of the tendon clamps were filled and after 9 min loading was initiated. Pilot test showed 9 min as ideal timepoint for the tendons being form fit frozen in the clamps to prevent slippage of the tendon tissue. All grafts were randomized for testing and load to failure tests were carried out at room temperature. An initial preload of 20 N was applied to ensure all fibers were evenly loaded. Grafts were then preconditioned from 10 to 50 N for 20 cycles at 20 mm/min to minimize soft tissue viscoelasticity before being loaded to failure at a speed of 20 mm/min. Load and displacement were recorded and ultimate load, ultimate elongation, and stiffness of the linear region of the load–displacement curve (ranging from 2.5 to 7.5 mm elongation) were determined from load–displacement data of load-to-failure testing for each graft. Failure mode was also recorded for each sample. Additionally, combined with specimen length and diameter stress-strain plots were derived from the load displacement data and ultimate stress, ultimate strain and the E-modulus was determined in the linear region (ranging from 5 to 15% strain) of the stress-strain plot of each graft.

### Statistical analysis

For statistical analysis and graphical representation, Microsoft Excel (Microsoft Corporation, Redmond, WA, USA) and IBM SPSS Statistics V28 (IBM Corporation, Armonk, NY, USA) were used. Testing data for normal distribution was performed using the Kolmogorov-Smirnov test.

Further data analysis of the normally distributed parameters was performed by the one-factor analysis of variance (ANOVA) with Bonferroni post hoc tests. Significance level was set to 0.05.

## Results

**Fig. 2:**
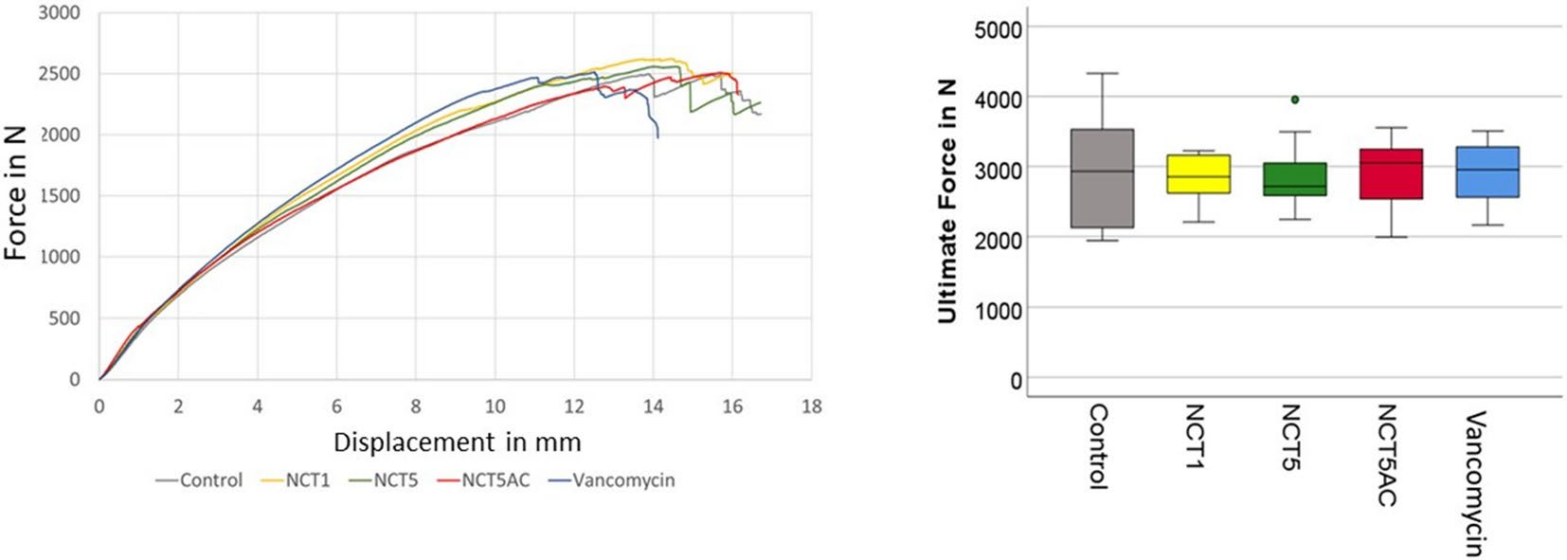
Exemplary force-displacement plots of the five groups (left) and boxplot of the ultimate force derived of the force-displacement plots showing the median and the quartiles (25% and 75%) of 10 samples for all five test groups (right)

The mean diameter of the CSA of all grafts was 5.7 ± 0.6 mm ranging from 5.0 to 7.0 mm, and the mean weight was 3.9 ± 0.7 g, with no statistically significant difference between the groups (Table 1). The force displacement graphs of all tested specimens had a similar shape (Fig. 2).

The mean ultimate load to failure of all grafts in all groups was 2887.7 ± 534.4 N (ranging from 1943 N to 4238 N) with mean values of the five test groups ranging from 2800.7 N to 2933.7 N (Table 1; Fig. 2) and analysis of variance revealed no significant differences between the groups (*p* = 0.98). The mean elongation at ultimate load was 13.62 ± 2.23 mm for all specimens, with similar elongations observed across all five groups (*p* = 0.80). The stiffness was determined from the linear range of all test specimens in the force-displacement graph for the elongation between 2.5 and 7.5 mm. It showed a mean of 245.0 ± 44.2 N/mm for all specimens and with no difference between groups (*p* = 0.90).

**Fig. 3:**
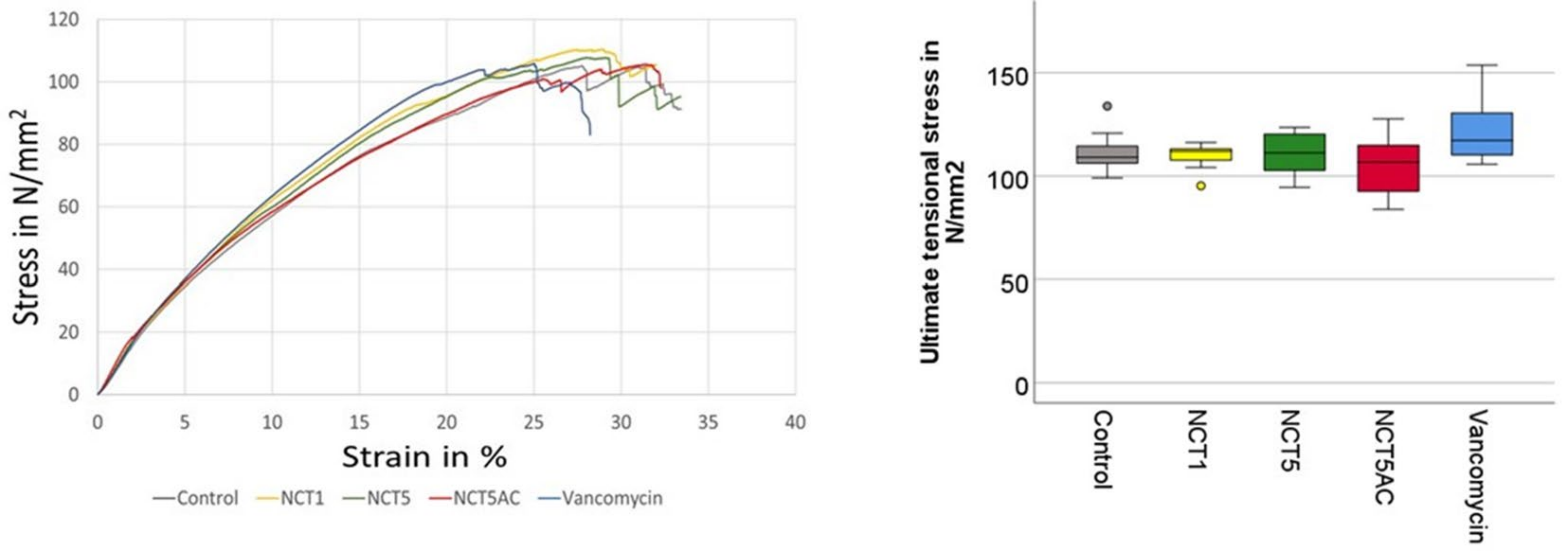
Exemplary stress-strain plots of the five groups (left) and boxplot of the ultimate tensional stress derived of the stress-strain plots showing the median and the quartiles (25% and 75%) of 10 samples for all five test groups (right)

The mean ultimate tensional stress was calculated with the CSA of each specimen and showed a mean of 111.8 ± 12.3 N/mm² for all specimens and ranged from 106.3 to 121.3 N/mm^2^ for all five groups (Table 1; Fig. 3) with no significant differences between groups (*p* = 0. 68). The strain at ultimate stress was calculated with the specimen length and its elongation at ultimate load. The mean strain at ultimate stress for all specimens was 27.3% with means of the ultimate strain of the five groups ranging from 25.7 to 27.9% (Table 1; Fig. 3). There were no significant differences between the five groups (*p* = 0.78). The elastic-modulus was determined from the linear range of all test specimens in the stress-strain graph in the strain range of 5 to 15%. Overall, the mean value of the elastic modulus of all five test groups was 477.4 ± 57.0 N/mm^2^ without any group difference (*p* = 0.57).

The stress-strain curves exhibited a similar linear increase up to approximately 20% strain, after which irreversible deformation started. This is indicated by a drop in tensional stress due to the rupture of the initial tendon fibers. In all test series, the failure mode was tendon rupture, occurring either transversely, longitudinally, at the midpoint of the tendon, or near the clamping area. No slippage of the tendons from the clamps was observed.

**Table 1:** Tendon characteristics and outcomes (average ± standard deviation of 10 samples each)

	Control	Vancomycin	NCT1	NCT5	NCT5AC
Weight (in g)	3.93 ± 0.89	3.61 ± 0.62	3.79 ± 0.57	4.02 ± 0.8	4.07 ± 0.7
Diameter (in mm)	5.8 ± 0.7	5.6 ± 0.5	5.7 ± 0.5	5.8 ± 0.5	5.9 ± 0.5
Ultimate load (in N)	2933.7 ± 813.6	2925.5 ± 425.8	2800.7 ± 376.6	2870.1 ± 541.7	2908.3 ± 541.7
Elongation at ultimate load (in mm)	13.8 ± 2.4	13.8.0 ± 2.4	13.6 ± 2.6	14.1 ± 1.8	12.9 ± 2.1
Stiffness (in N/mm)	246.7 ± 57.2	254.1 ± 47.3	234.4 ± 37.8	241.7 ± 47.8	248.1 ± 34.0
Ultimate tensional stress (in N/mm^2^)	111.1 ± 10.2	121.3 ± 14.8	109.7 ± 6.1	110.4 ± 10.6	106.3 ± 14.3
Strain at ultimate tensional stress (in %)	27.6 ± 4.8	27.9 ± 5.2	27.1 ± 53	28.1 ± 3.6	25.7 ± 4.2
Elastic modulus (in N/mm^2^)	482.8 ± 63.2	521.8 ± 36.8	459.8 ± 49.8	465.6 ± 59.8	456.8 ± 55.3

## Discussion

The key finding of this study is that pre-soaking bovine extensor tendons in different concentrations of NCT or vancomycin does not affect the tendons’ structural properties. The initial hypothesis was confirmed, as no statistically significant differences were observed in ultimate load to failure, ultimate elongation, maximum tension, stiffness, or elastic modulus.

Topical application of vancomycin to tendon grafts for decontamination prior to implantation has significantly reduced the incidence of PSA [3, 7, 8]. However, despite these promising clinical outcomes, little is known about potential adverse effects, such as chondrotoxicity or the long-term impact of intraarticular vancomycin application [9, 13]. A particular concern is the limited spectrum of vancomycin against Gram-positive bacteria and the potential for developing bacterial resistance, which could pose unforeseen challenges, particularly in cases of required revision surgery. Given the increasing prevalence of antibiotic-resistant bacteria, largely driven by overuse and inappropriate prophylactic strategies, the use of local antibiotics warrants careful reconsideration, and alternatives may be necessary in the future.

A possible alternative for infection control might be NCT. NCT demonstrates broad-spectrum antimicrobial activity without fostering resistance, has low toxicity, is naturally occurring within the body, and exhibits anti-inflammatory properties without systemic distribution [15, 16, 18, 20]. Due to its oxidizing and chlorinating mechanism of action inherent in active chlorine compounds, NCT and related compounds do not induce resistance in pathogens at least at therapeutic concentrations [21]. Moreover, strains resistant against antibiotics are similarly susceptible to NCT as strains susceptible to antibiotics [22]. These are general advantages of this class of antiseptics over antibiotics. Moreover, NCT has demonstrated favourable tolerability by chondrocytes in vitro, exhibiting lower cytotoxicity than povidone-iodine (PVP-I) and hydrogen peroxide (H₂O₂) when applied at low concentrations [23]. This reduced cytotoxic profile further supports its potential utility in orthopaedic surgery [23]. While there is extensive evidence supporting that vancomycin pre-soaking of ACL grafts does not negatively affect tendon properties, this study is the first to investigate the impact of NCT on the structural properties of tendons [24, 25]. Addition of ammonium chloride (NH_4_Cl) to NCT enhances its microbicidal effect by forming the more lipophilic monochloramine (NH_2_Cl) in equilibrium, which may be of advantage when rapid decontamination of implant grafts is desired [18, 19].

Therefore, we additionally tested a combination of both. The present data clearly demonstrate that, when compared directly with vancomycin or control (0.9% saline solution), tendon properties remain unaffected following decontamination in NCT-soaked gauze for 10 min. This effect remains unchanged regardless of the concentration used, indicating that NCT and its combination with NH_4_Cl does not compromise the structural integrity of the tendons during the decontamination process.

Similar results were reported in previous animal studies using vancomycin. No statistically significant differences in tendon properties were observed when comparing this antibiotic and saline on porcine flexor digitorum profundus tendon properties [14]. Likewise, neither saline, vancomycin, nor buffered vancomycin adversely affected the time-zero material properties of bovine bone-patellar-tendon-bone grafts [24]. These results have also been confirmed in human semitendinosus grafts, where no statistically significant alterations in biomechanical properties were found between untreated grafts and those pre-soaked in vancomycin [25].

With the hierarchical structure of tendons composed of multiple strands of tropocollagen forming a fibril, multiple fibrils a fiber, multiple fibres a fascile and multiple fascile a tendon, a tendon is composed of 70 to 90% of collagen primarily aligned in the tendons tensile loading direction. Orthogonal to adjacent fibrils covalent cross links are present which also contribute to the mechanical properties in fiber directions [26]. These interfibrilar cross links are composed of proteoglycan molecules bound to collagen fibrils and two linked glycosaminoglycans (GAGs) connecting to the proteoglycans [27]. While the main mechanical competence is in the collagen fibres and their crimp pattern, the contribution of the cross links in particular in the direction of the collagen fibres is still discussed [28]. In literature no mechanical effect is reported for soaking tendons in vancomycin, therefore it might be assumed that it does not alter the hierarchical structure of tendons or the interfibrilar cross linking. This is in accordance with the specific mechanism of action of the antibiotic, which is inhibition of cell wall synthesis by binding to d-alanyl-d-alanine and therefore blockage of transpeptidases, while collagen mainly consists of glycine and proline [29, 30].

On a molecular level, NCT as an active chlorine compound exerts several chemical reactions. It rapidly oxidizes thio groups (sulfhydryls and thioethers), which leads to a loss of oxidation capacity [31–34]. Good examples are the immediate inactivation of NCT by addition of sodium thiosulphate or a mixture of methionine and histidine. Loss of oxidation capacity is also valid for chlorination of aromatic compounds, for instance phenol, whereby the reaction is slower [31]. Finally, chlorination of amino groups occurs with formation of the corresponding chloramines in equilibrium, which is not connected with a loss of oxidation capacity. These reactions sufficiently explain the microbicidal activity of NCT and its cytotoxicity in cell culture. As a mild oxidant, however, NCT does not react with amides and imides and further groups, which is in strong contrast to active chlorine compounds with moderate or strong activity such as chloramine T and hypochlorite [31, 32, 34]. This explains the high tolerability of NCT by tissue in vivo, even in highly sensitive body regions such as the eye or lower airways [18, 20]. The prevailing glycins and prolins in collagen form amides in the protein, which are not attacked by NCT. Therefore, the low reactivity of NCT and the limited number of its reaction partners is in accordance with the maintenance of the function of tendons after treatment with this antiseptic.

Antiseptics in general do not seem to impact the function of tendons when they are used for decontamination. Chlorhexidine at a high concentration of 2% and a volume of 3 L did not influence biomechanics of bovine superficial digital flexor tendons in an in vitro study [35]. This was confirmed with human patellar tendon allografts soaked for 30 min in 4% chlorhexidine [36]. Preservation of demineralized bone matrix with polyvinylpyrrolidone-iodine (PVP-I) used as an allograft in a thigh muscle pouch model of nude mice in vivo was superior to gamma irradiation sterilization [37]. This was expressed in better ectopic formation and higher mechanical strength. In another in vivo model in rabbits, autologous tendon transplantation after treatment with 100 µM PVP-I for 30 min compared to normal saline for 30 min resulted in improved healing and better biomechanical properties of the grafts [38]. Because of the even lower cytotoxic potential of NCT compared to these antiseptics [23, 39], the absence of an impact on the biomechanical properties of tendons in the present study is not surprising. The good tolerability of NCT even in highly sensitive body regions renders it a promising compound among others for irrigation of the operation field subsequent to implantation of grafts to prevent infection or for treatment of septic or rheumatoid arthritis [15, 16, 40].

Several potential limitations of the current study should be acknowledged. First, as bovine extensor tendons were used as grafts, it is uncertain whether the results can be directly extrapolated to autologous ACL grafts typically used in clinical practice. Moreover, it is important to note, that the biomechanical properties of tendons are influenced by several factors, including the animal species, age, whether the tendons are from a living donor or cadaver, preservation methods, and tendon diameter. Contrary to expectations, whether the tests are conducted on fresh or frozen-thawed tendons does not significantly affect the results. Therefore, it is important to note that freezing tendons has little to no impact on their mechanical properties, as confirmed by previous studies [41, 42]. Lastly, the sample size of *n* = 10 per group, might not be sufficient for a definitive assessment. However, the sample size was considered adequate, because of the small dispersion and normal distribution of the data. By increasing the sample size to 100 and more in each of the five test groups a statistical significance might be reached, while the clinical difference is questionable.

## Conclusions

Wrapping tendon grafts in NCT or vancomycin has no immediate adverse effects on their structural properties, even at high concentrations. From a biomechanical standpoint, NCT does not appear to have any negative effects on the graft, suggesting that it may be a viable alternative to vancomycin for use in clinical applications.

## Data Availability

No datasets were generated or analysed during the current study.
